# V-ATPase Dysfunction in the Brain: Genetic Insights and Therapeutic Opportunities

**DOI:** 10.3390/cells13171441

**Published:** 2024-08-28

**Authors:** Antonio Falace, Greta Volpedo, Marcello Scala, Federico Zara, Pasquale Striano, Anna Fassio

**Affiliations:** 1 Pediatric Neurology and Muscular Diseases Unit, IRCCS Istituto Giannina Gaslini, 16147 Genoa, Italy; pstriano@unige.it; 2 Department of Neurosciences, Rehabilitation, Ophthalmology, Genetics, Maternal and Child Health, University of Genoa, 16132 Genoa, Italy; greta.volpedo@edu.unige.it (G.V.);; 3 Medical Genetics Unit, IRCCS Istituto Giannina Gaslini, 16147 Genoa, Italy; 4 Department of Experimental Medicine, University of Genoa, 16132 Genoa, Italy; 5 IRCCS, Ospedale Policlinico San Martino, 16132 Genoa, Italy

**Keywords:** v-ATPse, lysosomal dysfunction, neurodevelopmental disorders, neurodegeneration

## Abstract

Vacuolar-type ATPase (v-ATPase) is a multimeric protein complex that regulates H^+^ transport across membranes and intra-cellular organelle acidification. Catabolic processes, such as endocytic degradation and autophagy, strictly rely on v-ATPase-dependent luminal acidification in lysosomes. The v-ATPase complex is expressed at high levels in the brain and its impairment triggers neuronal dysfunction and neurodegeneration. Due to their post-mitotic nature and highly specialized function and morphology, neurons display a unique vulnerability to lysosomal dyshomeostasis. Alterations in genes encoding subunits composing v-ATPase or v-ATPase-related proteins impair brain development and synaptic function in animal models and underlie genetic diseases in humans, such as encephalopathies, epilepsy, as well as neurodevelopmental, and degenerative disorders. This review presents the genetic and functional evidence linking v-ATPase subunits and accessory proteins to various brain disorders, from early-onset developmental epileptic encephalopathy to neurodegenerative diseases. We highlight the latest emerging therapeutic strategies aimed at mitigating lysosomal defects associated with v-ATPase dysfunction.

## 1. Introduction: v-ATPase Structure and Function

**Figure 1:**
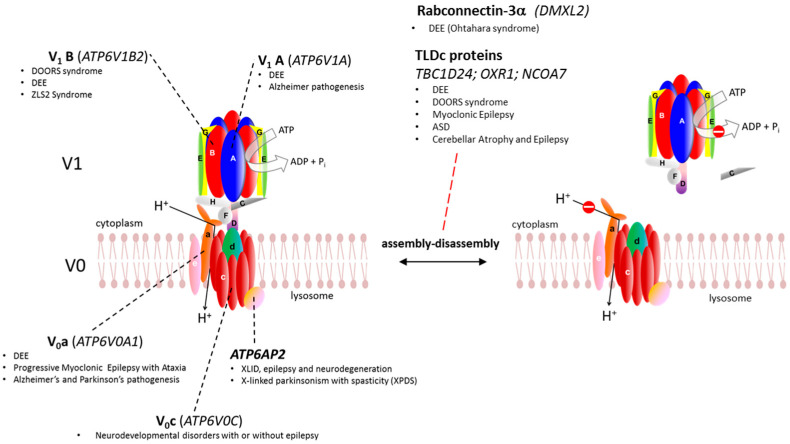
v-ATPase assembly/disassembly. Genetic defects and associated brain diseases are marked for the subunits, accessory protein, and assembly/disassembly regulators. **Legend**: ATP6AP2, ATPase H-transporting lysosomal accessory protein 2; ASD, autism spectrum disorder; DEE, developmental and epileptic encephalopathy; DOORS, deafness, onychodystrophy, osteo-dystrophy, retardation and seizures; XLID, X-linked intellectual disability; ZLS2, Zimmermann–Laband syndrome 2.

Rotary adenosine triphosphatases (ATPases) play key roles in energy conservation, transmembrane transport, acidification of intracellular compartments, and pH homeostasis. They are divided into three classes based on their structure and the type of ion they transport [F-ATPases (e.g., mitochondrial H^+^-ATPase), P-ATPases (e.g., Ca^2+^-ATPase, Na^+^, K^+^-ATPase), and V-ATPases (e.g., lysosomal H^+^-ATPase)]. Upon ATP hydrolysis, vesicular or vacuolar-type ATPases (v-ATPases) mediate hydrogen ion transport. In mammals, two different domains operating with a rotary mechanism make up this highly conserved multimeric complex: the V1 domain, composed of eight cytosolic subunits, binds and hydrolyses ATP in the cytosol, while the V0 domain, composed of seven transmembrane subunits, forms the hydrogen pore [1] (Figure 1). Upon ATP hydrolysis, V1 drives the movement of a central rotary complex, which results in proton translocation through the V0 domain. V0 and V1 can reversibly associate and dissociate to regulate the activity of the proton pump, which can be modified by several factors, including nutrient availability, hydrogen ion concentration, growth factors, and kinases [2,3,4]. Dissociation or failed assembly of the V1 and V0 domains hinder ATP-driven proton pumping, leading to a lack of proton transport across membranes. Maintaining and regulating the pH across the membrane is important in various cellular processes, such as membrane trafficking, protein degradation, autophagy, and small molecule transport [1,5]. In addition, v-ATPases are molecular hubs for sensing and/or transmitting signals through the mTOR, WNT, and NOTCH pathways [6]. Upon binding with the v-ATPase, mechanistic target of rapamycin complex 1 (mTORC1) localizes to the lysosomal surface and promotes the mTOR pathway via the Ragulator–Rag protein complex, thus modulating transcription factor EB (TFEB)-dependent gene cascade [7,8,9,10,11,12].

In humans, a redundant set of subunits is encoded by 22 autosomal genes, enabling the assembly of distinct v-ATPase complexes composed of subunits expressed in a tissue-specific manner, ultimately resulting in different degrees of regulated acidification [13,14]. The selective expression of specific accessory proteins, such as ATPase H-transporting lysosomal accessory protein 2 (ATP6AP2), is important for directing v-ATPase to distinct tissue types and specific membranes [15].

Due to its key role in cellular homeostasis, pathogenetic variants in v-ATPases-related genes are associated with various disorders spanning from osteoporosis to renal tubular acidosis and neurological diseases [5].

The v-ATPase complex is expressed at high levels in neurons, where it plays additional and unique roles, such as neurotransmitter loading into synaptic vesicles [16,17]. In this context, alterations in genes encoding v-ATPase subunits or their regulators have recently been described in different neurological disorders and in aging.

Here we discuss genetic and functional evidence linking v-ATPase subunits and the most relevant v-ATPase accessory proteins in the pathogenesis of different brain disorders, spanning from early-onset developmental epileptic encephalopathy to neurodegenerative conditions (Figure 1 and Table 1). The most recent emerging findings on therapeutical strategies for v-ATPase-related brain disorders are also discussed.

## 2. v-ATPase Gene Variants in Brain Disorders

### 2.1. Genes Encoding Subunits of the V1 Domain

The V1 cytosolic domain, composed of eight distinct subunits (A–H), has the function of binding and hydrolyzing ATP [1] (Figure 1). Genetic mutations of this domain can compromise enzymatic activity, as well as impairing subunit interactions and pump rotation. The core of the V1 domain is composed of a protein ring with three couples of alternate V1A and V1B subunits (Figure 1). Altered function of the V1 domain in the brain leads to altered pH homeostasis and ultimately to the development of neurological disorders as consequence of autophagic failure and synaptic defects. In particular, mutations in *ATP6V1A*, encoding V1A subunit, can promote developmental and epileptic encephalopathy (DEE) and its downregulation was associated with late-onset Alzheimer’s disease (AD), while mutations in *ATP6V1B2*, encoding the V1B subunit, mediate DEE, deafness, onychodystrophy, osteodystrophy, intellectual disability, and seizure (DOORS) syndrome, and Zimmermann Laband Syndrome (ZLS) (Figure 1).

### 2.2. De Novo Mutations of the ATP6V1A Gene Cause DEE

Recessive mutations in the *ATP6V1A* gene, coding for the V1A subunit, were first identified in patients presenting with cutis laxa, dysmorphic features, and seizures [18]. More recently, several de novo missense mutations in *ATP6V1A* have been described in patients with DEE of variable severity, with symptoms ranging from moderate intellectual disability (ID) and seizures to early-onset DEE with premature lethality [19,20]. The affected individuals (age range: 6 weeks–22 years old) primarily showed a spectrum of neurodevelopmental phenotypes of different degrees of severity, including small head size, hypotonia, ID, multiple seizure types (mostly infantile spasms), and motor disorders. At a mean age of 7 years old, the clinical picture included early lethal encephalopathies with rapidly progressive and massive brain atrophy, severe DEE, and ID with epilepsy. Less severe outcomes (25% incidence) were represented by moderate ID and variable epilepsy. Additionally, less than half of the patients showed microcephaly and amelogenesis imperfecta/enamel dysplasia. Brain MRIs demonstrated hypomyelination, as well as generalized and progressive atrophy in two-thirds of the patient cohort [20].

Additional evidence for the deleterious effects of *ATP6V1A* variants was provided by structural modelling, thanks to the recently solved atomic structure of the mammalian brain v-ATPase [21,22]. Thirteen of the *ATP6V1A* mutations described in this study were located on ATP-binding or catalytic sites at the interface of subunits A and B, indicating that they may directly impair enzymatic activity. Modelling of the remaining variants suggests that some can hinder v-ATPase rotation or subunit interactions, while others may alter protein expression and stability [20]. Taken together, these observations highlight the importance of the V1 subunit A, and ultimately of a functional v-ATPase, for correct neurodevelopment.

In vitro functional experiments further consolidated the pathogenetic role of *ATP6V1A* variants and are consistent with lysosomal impairment and loss of v-ATPase function in patients’ cells. Fibroblast cultures from two *ATP6V1A*-related DEE patients showed decreased expression of the lysosomal marker LAMP1 and LysoTracker staining, as well as increased organelle pH. In contrast, patients’ cells with milder symptoms exhibited an opposite phenotype, suggesting that v-ATPase ‘gain-of-function’ may elicit milder phenotypes [20]. Ultrastructural analysis in both patients’ derived fibroblasts and induced pluripotent cell-derived neurons revealed ultrastructural defects in autolysosomes, which displayed accumulation of non-degraded material of largely unknown composition [20]. When expressed in postsynaptic rodent neurons, *ATP6V1A* pathogenetic variants impaired neurite development and excitatory synaptic connections [19]. Additional heterozygous variants have been described in patients with favorable epilepsy resulting in ATP6V1A loss of expression [23,24].

### 2.3. ATP6V1A and Neurodegeneration

A multi-omic molecular analysis of late-onset Alzheimer disease (LOAD) across four brain regions in 364 donors with varying cognitive and neuropathological phenotypes identified *ATP6V1A* as a top key regulator of the most dysregulated neuronal subnetwork in LOAD [25,26]. Furthermore, *ATP6V1A* was consistently downregulated in multiple brain regions in individuals with dementia, including those in the early stages of the disease [27]. The *ATP6V1A* gene was also identified as a disease driver in a parallel study characterizing the molecular heterogeneity of Alzheimer disease (AD), by using an integrative network approach on 1543 transcriptomes across five brain regions in two AD cohorts [25].

Experimental in vitro approaches demonstrated that CRISPR/Cas9-mediated *ATP6V1A* downregulation in human iPSC-derived excitatory neurons impairs electrical activity and promotes a selective loss of the synaptic vesicle markers SYN1 and vGLUT1, suggestive of pre-synaptic dysfunction. Neuronal excitability was further compromised by the presence of the toxic amyloid-beta (Aß42) peptide, which has a well-known association with AD pathogenesis [25]. In vivo studies in a Drosophila model genetically ablated for the two orthologs of *ATP6V1A* showed that reduced neuronal expression was sufficient to impair motor function. These changes were accompanied by diminished mRNA levels of synaptic genes, corroborating the synaptic dysfunction found in hiPSC-derived neurons. Aß42 expression alone was also associated with reduced expression of the *ATP6V1A* orthologs in the fly brain [25]. Furthermore, in a recent study, Esposito et al. showed that *Atp6v1a* silencing in rat hippocampal neurons affected synaptogenesis and plasticity, as well as lysosomal and autophagic function [28]. Taken together, these pre-clinical and clinical studies highlight the role of the *ATP6V1A* gene as a driver for synaptic dysfunction and subsequent neuronal loss, and a potential therapeutic target to prevent neurodegeneration.

### 2.4. ATP6V1B2 De Novo Truncating Variant in DOORS Syndrome

Loss of *ATP6V1B2*, a gene coding for the Β2 subunit of the v-ATPase V1 cytosolic domain, is non-compatible with life. In fact, complete deletion of *ATP6V1B2* results in embryonic lethality prior to organogenesis in mice (https://www.mousephenotype.org/data/genes/MGI:109618 (accessed on 5 July 2024). In humans, a recurrent truncating variant in *ATP6V1B2* has been associated with DOORS syndrome. Beauregard-Lacroix et al. reported the *ATP6V1B2* variant c.1516C>T.; p.Arg506* in nine individuals from eight unrelated families with DOORS syndrome [29]. To characterize the function of the *ATP6V1B2* p.R506* variant in brain circuits, a knock-in *Atp6v1b2*emR506* mouse model was generated by CRISPR/Cas9-mediated gene editing [30]. No spontaneous seizures were recorded in homozygous *Atp6v1b2*emR506* mice; however, continuous intracranial video-EEG recordings revealed that P90-P110 *Atp6v1b2*emR506* mutant mice presented frequent interictal epileptic activity and stimuli-induced seizures. In addition, *Atp6v1b2*emR506* mice displayed seizure susceptibility to a range of pro-convulsive (Pentylenetetrazole) triggers [30], highlighting the role of this subunit in proper v-ATPase functioning and brain homeostasis.

### 2.5. ATP6V1B2 Recurrent De Novo Missense Variants in Zimmermann-Laband Syndrome

Zimmermann–Laband syndrome (ZLS) is a developmental disorder characterized by facial dysmorphism with gingival enlargement, ID, hypoplasia or aplasia of nails and terminal phalanges, as well as hypertrichosis. In 2015, Kortum et al. identified a recurrent missense change in *ATPV1B2* (NM_001693.3 c.1454G-C, p.Arg485Pro) in two unrelated ZLS patients [31]. Bioinformatic analysis showed that the arginine-to-proline substitution in ATP6V1B2 was predicted to perturb inter-subunit interactions within the V1 subcomplex by destabilizing the C-terminal segment of the B subunit, which is part of the A-B interface and interacts with the E subunit [31]. Destabilization of the V1 domain leads to v-ATPase dysfunction, highlighting the crucial role for appropriate pH regulation and v-ATPase-mediated signaling during brain development.

### 2.6. ATP6V1B2 Pathogenetic Variants in Epileptic Syndromes and DEE

In 2017, Popp et al., reported an heterozygous change (c.1120G>C, p.Glu374Gln) in *ATPV1B2* in an individual with severe ID, hypotonia, microcephaly, and seizures [32]. Subsequently, Shaw et al. described a large Polish family with relatively mild gingival and nail problems, no phalangeal hypoplasia, and with generalized epilepsy, harboring a novel dominant missense variant (c.1192C>G, p.Leu398Val) in *ATP6V1B2* [33]. More recently, de novo variants in *ATP6V1B2* have also been found in DEE patients. Inuzuka et al. reported the case of an infant with severe epileptic encephalopathy, microcephaly, and profound developmental delay associated with a novel de novo loss-of-function variant (c.1465A>T, p.Lys489*) in *ATP6V1B2*, diagnosed by whole-exome sequencing [34]. Furthermore, exome sequencing identified a novel de novo variant in *ATP6V1B2* (c.973G>C, p.Gly325Arg) in a patient affected by global developmental delay, skeletal abnormalities, and epileptic encephalopathy featuring Lennox–Gastaut syndrome [35]. Taken together, these data identify a role for the V1B subunit in neuronal excitability and seizures.

### 2.7. Genes Encoding Subunits of the V0 Domain

The V0 transmembrane domain forms the hydrogen pore and consists of seven distinct subunits [1] (Figure 1). Variants in the V0A subunit can promote DEE and myoclonic epilepsy with ataxia, while V0C variants lead to neurodevelopmental disorders with or without epilepsy (Figure 1). Moreover, defects in V0A subunit function drive physiopathological events in early-onset AD.

### 2.8. De Novo and Biallelic ATP6V0A1 Variants Cause DEE and Progressive Myoclonic Epilepsy with Ataxia

*ATP6V0A1* encodes for the brain-enriched isoform of the A subunit and is part of the CLEAR (coordinated lysosomal expression and regulation) network of genes regulated by TFEB [36]. Recently, two studies have reported various de novo and biallelic *ATP6V0A1* variants as major causes of DEE [37,38]. Biallelic variants in this gene have been identified also in five patients affected by early-onset progressive myoclonus epilepsy with ataxia [38]. Functional studies performed in the *Caenorhabditis elegans* model showed that the recurrent DEE pathogenetic variant p.Arg740Glu leads to failure of lysosomal activity by directly impairing acidification of the endo-lysosomal compartment, thus causing autophagic dysfunction and severe developmental defects [38]. Neuro2a cell lines stably expressing the p.Arg740Glu variant displayed impaired protonation and decreased level of the lysosomal enzyme Cathepsin D, accompanied by the accumulation of the autophagosomal marker LC3-II [38]. Moreover, Aoto et al. developed a knock-in mouse model harboring either the human p.Arg740Glu or the p.Ala505Pro variant, also associated with DEE [37]. *Atp6v0a1* R740Q/R740Q mice do not survive the embryonic stage, suggesting that this mutation leads to a complete v-ATPase loss-of-function, which is not compatible with life. *Atp6v0a1* A505P/A505P mice survived for 2 weeks and displayed impaired motor function and ataxia [37,39]. *Atp6v0a1* A505P/A505P brain staining revealed reduced numbers of layer V neurons and impaired synaptogenesis. Furthermore, the decreased levels of the Atp6v1a protein in the *Atp6v0a1* A505P/A505P model suggested that the Ala505Pro variant affects the stability of the v-ATPase complex [37]. Investigations of lysosomal and autophagic function revealed increased neuronal cell death, abnormal cellular distribution of lysosomes, decreased cathepsin D activity, as well as autophagosome and lysosome accumulation in the *Atp6v0a1* A505P/A505P mouse brain [37]. These phenotypes were accompanied by a concomitant decrease of mTORC1 activity. Furthermore, electrophysiological recordings in *Atp6v0a1* A505P/A505P cultured neurons demonstrated a reduction in the amplitude and frequency of both miniature excitatory and inhibitory postsynaptic currents, suggestive of lowered neurotransmitter content in the synaptic vesicles and decreased neurotransmitter release [37,39]. Taken together, these studies highlight the pathogenic role of *ATP6V0A1* variants during neurodevelopment, impacting both the number and function of different neuronal populations.

### 2.9. ATP6V0A1 and Neurodegeneration

Pathogenetic variants in *Presenilin 1* (*PS1*) are the most common cause of early-onset familial AD (FAD) [40]. *PS1* encodes for a ubiquitous transmembrane protein showing complex roles in cell adhesion, apoptosis, neurite outgrowth, calcium homeostasis, and synaptic plasticity [41]. Following cleavage at the endoplasmic reticulum, PS1 participates in the gamma secretase complex, which mediates the intramembranous cleavage of amyloid-beta precursor proteins (APPs) [41]. Disease-causing *PS1* mutations increase the generation of the neurotoxic amyloid-β peptide from APP, a classical hallmark of AD. PS1 facilitates the maturation and delivery of the v-ATPase V0a1 subunit to the lysosome [41]. Therefore, lysosomal acidification and autophagy are severely impaired in the brain of *PS1*-deficient mice. Of note, autophagic failure and v-ATPase mistargeting were also observed in fibroblasts from patients with FAD harboring deleterious variants in *PS1* [41]. Constitutively, phosphorylated APP selectively interacts with the cytoplasmic domain of the V0a1 subunit, thus preventing association of the V0/V1 complex in vitro and in vivo [42]. v-ATPase lysosomal acidification was defective and v-ATPase assembly disrupted in fibroblasts from patients with Down syndrome (DS) [42], the congenital genetic disorder responsible for the most prevalent form of early-onset AD [43]. These findings were also confirmed in the brain tissue from a DS animal model (*Ts2* mouse) and from a DS patient [41]. Reducing APP phosphorylation can rescue v-ATPase function and lysosome acidification in DS fibroblasts and in the animal model [39]. Lastly, V0a1 subunit dysfunction can lead to lysosomal defects underlying neurodegenerative conditions, such as *LRRK2*-linked PD [44] and *CLN1*-associated disorders [45], highlighting the importance of this subunit in maintaining healthy brain functions.

### 2.10. De Novo Heterozygote ATP6V0C Variants in Patients with Neurodevelopmental Disorders with or without Epilepsy

*ATP6V0C* encodes for the 155-amino acid c-subunit of the V0 domain which, along with the c′′ subunit (encoded by *ATP6V0B*), forms the intramembrane c-ring that facilitates the movement of protons across the membrane [46]. Mucha et al. described a 16p13.3 microdeletion resulting in simultaneous haploinsufficiency of *TBC1D24*, *ATP6V0C*, and *PDPK1*, underlying a neurological syndrome characterized by microcephaly, developmental delay, intellectual disability, and epilepsy [47]. Interestingly, despite the presence of the epilepsy gene *TBC1D24* in the genomic deletion, *ATP6V0C* haploinsufficiency was proposed as the primary contributor to the clinical features of the 16p13.3 microdeletion syndrome [48]. The identification of a de novo stop-loss *ATP6V0C* variant in an individual with epilepsy and ID further corroborated this hypothesis [49,50,51]. More recently, Mattison et al. identified heterozygous *ATP6V0C* missense variants in 27 patients with a novel syndrome of developmental delay, epilepsy, and ID [52] (OMIM# 620465). Generalized tonic–clonic, focal, atonic, and myoclonic seizures were reported in 19 patients of the cohort with a mean age onset of 24.6 ± 8.0 months. Common MRI findings included agenesis/hypoplasia of the corpus callosum or cerebellar vermis and delayed myelination. In silico modelling suggested that the patient variants interfered with the interactions between the *ATP6V0C* and *ATP6V0A* subunits during ATP hydrolysis [52]. The deleterious effects of *ATP6V0C* variants were also assessed by functional analyses in *Saccharomyces cerevisiae* and in invertebrate models. Three selected variants modelled in *Caenorhabditis elegans* led to motor dysfunction, as well as reduced growth and survival [52]. Knockdown of *Dmel*\*Vha16–3*, the *ATP6V0C* orthologous in *Drosophila*, resulted in seizure-like behavior and pretreatment of these larvae with a variety of established antiepileptic drugs resulted in significant reductions in recovery time [52], consistent with the hypothesis that haploinsufficiency of *ATP6V0C* contributes to seizures in multiple disease models.

## 3. The Pathogenetic Role of V-ATPase Accessory Proteins

v-ATPase is accompanied by accessory proteins which can enhance stability and regulate the function of the pump. The ATP6AP2 accessory protein is associated with the transmembrane domain and plays a role in v-ATPase assembly and lysosome acidification. Furthermore, DMXL2 plays a role in V0/V1 assembly and signal transduction. Mutations in their encoding genes can lead to neurodegenerative disorders, such as Parkinson’s disease (*ATP6AP2*) and Ohtahara syndrome (*DMXL2*). More recently, the modulatory roles of Tre2/Bub2/Cdc16 (TBC), lysin motif (LysM), and domain catalytic (TLDc) protein family members on v-ATPase function have also emerged. TLDc family genes include *TBC1D24*, *NCOA7*, and *OXR1*, which have been extensively associated with neurological conditions.

### 3.1. De Novo ATP6AP2 Variant in X-Linked Intellectual Disability (XLID), Epilepsy and Neurodegeneration

The *ATP6AP2* (ATPase, H+ transporting, lysosomal accessory protein 2) gene on Xp11.4 encodes for a v-ATPase accessory protein firstly identified for its role as a (pro)renin receptor in the renin–angiotensin system [53]. *ATP6AP2* is highly expressed in the CNS from the earliest stages, and functional studies performed in yeast and flies suggest that ATP6AP2 participates in the assembly of the v-ATPase proton pore in the endoplasmic reticulum [54]. ATP6AP2 plays a role in protein degradation and regulation of different signaling pathways involved in brain development and synaptic transmission. It is also predicted to enable signaling receptor activity [55,56].

A hemizygous silent mutation in *ATP6AP2* was identified in a putative exonic splicing enhancer site in a kindred of seven males [57,58]. These patients featured global developmental delay apparent from infancy and severe progressive neurologic decline with abnormal movements, spasticity, and seizures. The reported mutation resulted in inefficient inclusion of exon 4 of *ATP6AP2* gene [58]. Subsequently, a hemizygous 2-bp deletion in intron 3 of the *ATP6AP2* was identified in a male infant with a neurodevelopmental disorder and fulminant degeneration [59]. In patient-derived neurons, loss of *ATP6AP2* results in premature and/or ectopic differentiation, as well as accumulation of abnormal vacuoles and decreased spontaneous activity [59]. These defects were associated with severe deficiency in lysosomal acidification and protein degradation, thus leading to neuronal cell death [59]. Telencephalic *Atp6ap2* conditional knockout male mice showed a postnatal growth delay and died at around 4 weeks of age as a consequence of premature progenitor differentiation and the subsequent fulminant death of newborn neurons [59]. These findings were recapitulated in the fly model, suggesting that a loss of ATP6P2 leads to a lack of v-ATPase activity, impairment of autophagy, and severe neurodegeneration mimicking the pathogenesis observed in human mutations [59,60]. Altogether, these findings highlight the importance of v-ATPase accessory proteins in maintaining proper brain function.

### 3.2. Altered Splicing of ATP6AP2 Causes X-Linked Parkinsonism with Spasticity

In affected members of a family with X-linked parkinsonism with spasticity (XPDS), originally reported by Poorkaj et al. and Korvatska et al., identified a pathogenetic splice variant in the *ATP6AP2* gene, resulting in an internal deletion of 32 amino acids in the ATP6AP2 protein [61,62]. Postmortem patient brain tissue showed decreased ATP6AP2 levels in the frontal cortex and striatum, as well as a massive accumulation of p62 in the striatum, suggesting impaired autophagy and a defect in lysosome-mediated protein degradation [61]. In vitro *ATP6AP2* knockdown in a heterologous system led to v-ATPase pump dysfunction and to the accumulation of autophagosomes and autolysosomes [62]. More recently, Edelman et al. demonstrated that such internal deletion resulted in a 50% decrease of full-length ATP6AP2 in neuroprogenitor cells derived from XPDS patients, leading to the downregulation of networks related to neuronal fate commitment and axon guidance [63]. Taken together, these results highlight the sensitivity of brain cells to *ATP6AP2* gene expression levels and propose a link between PD and dysregulation of autophagy and protein degradation, mediated by v-ATPase activity.

### 3.3. Biallelic DMXL2 Variants Cause Ohtahara Syndrome with Progressive Course

The *DMXL2* gene encodes for rabconnectin-3α, a member of the WD40 repeat (WDR) protein family that is highly expressed in the brain. Rabconnectin-3α interacts with the synaptic vesicle (SV)-associated G-protein Rab3A at the synapse and is strictly involved in the assembly and incorporation of v-ATPase into SVs through its Rav1p domain, homologous to the yeast v-ATPase regulator Rav1p [64,65,66,67]. Loss of DMXL2 is associated with impaired v-ATPase activity in multiple experimental models [68,69,70,71], while biallelic mutations have been found in patients from three unrelated families with severe and rapidly progressing DEE associated with Ohtahara syndrome and premature death [72]. An additional homozygous variant in *DMXL2* has been reported in a patient with consanguineous parents and presenting DEE and macrocephaly [73]. *DMXL2*-related DEE manifested from the first day of life and was associated with deafness, mild peripheral polyneuropathy, and dysmorphic features. Repeated brain MRI investigations from the first months to the first years of life revealed progressive moderate brain shrinkage with leukoencephalopathy [72]. Functional studies performed in *DMXL2* patient fibroblasts showed enhanced LysoTracker signal associated with decreased endo-lysosomal markers and impairment of degradative processes. These defects were accompanied by impaired autophagy, revealed by diminished LC3II signal and accumulation of the autophagy receptor p62, with morphological alterations of the autolysosomal structures [72]. Altered lysosomal homeostasis and defective autophagy were recapitulated in *Dmxl2*-silenced mouse hippocampal neurons, which exhibited impaired neurite elongation and synaptic loss. Complete loss of *Dmxl2* is lethal at the embryonic stage in mice [66,67], whereas heterozygous *DMXL2* mice show macrocephaly and corpus callosum dysplasia [74], uncovering the relevance of early v-ATPase regulation in brain development and function.

### 3.4. Neurodevelopmental Disorders Associated with TLDc Domains Encoding Genes

Genes encoding TLDc proteins are associated with multiple neurodevelopmental disorders, although the physiopathological bases have not been fully elucidated. Human proteins sharing the TLDc domain include OXR1, NCOA7, TBC1D24, mEAK7, and TLDC2 and have been recently defined as a new class of V-ATPase-associated proteins [75]. Proteomic analyses of v-ATPase-associated proteins revealed that NCOA7 and OXR1 bind to the B1 subunit isoform of the V-ATPase [71]. Subsequent studies extended such interaction to all five members of the TLDc family and to additional cytosolic V1 subunits [76,77]. Castroflorio et al. showed that in primary cortical neurons NCOA7 depletion prevented v-ATPase holoenzyme assembly thus resulting in lysosomal deacidification and autophagic failure [76]. In contrast, Oot et al. showed that four purified human TLDc proteins (OXR1, NCOA7, TBC1D24, and TLDC2) inhibit V-ATPase by inducing its disassembly in vitro, whereas mEAK7 enhances v-ATPase activity [78]. Thus, the specific role of the different TLDc proteins on the dynamics of v-ATPase assembly/disassembly needs to be determined in a physiological context.

Amongst all TLDc domain-containing genes, *TBC1D24* is the most interesting in the context of this review, due to its role in the pathogenesis of a wide spectrum of neurological conditions, extending from benign idiopathic epilepsies to severe DEEs [79,80,81,82,83,84,85,86]. Interestingly, the clinical phenotype of *TBC1D24* biallelic variants may overlap with that of *ATP6V1B2* mutations, as both genes represent the major genetic cause of DOORS syndrome [87,88], implying a physiological link between TBC1D24 and v-ATPase function. At the cellular level, TBC1D24 impairment has been associated with neuronal phenotypes ranging from early developmental defects to synaptic dysfunction [89,90,91]. TBC1D24 is required for SVs and receptor trafficking [91,92,93], suggesting that the interplay between TBC1D24 and v-ATPase may occur at the synaptic compartments. However, further studies are needed to define the precise regulatory role of TBC1D24 in regulating v-ATPase function in the brain.

In addition to *TBC1D24*, two other TLDc domain-containing genes, *NCOA7* and *OXR1*, have been recently associated with neurodevelopmental disorders. The NCOA7 protein interacts specifically with the cytoplasmatic V1 subunits of the v-ATPase and is required for its proper assembly and activity in the brain [76]. A homozygous nonsense mutation in *NCOA7* was characterized in a recessive case of autism spectrum disorder [94]. Neurons lacking NCOA7 exhibit altered development accompanied with defective lysosomal formation and function. The *Ncoa7* deletion animal model exhibited abnormal neuronal patterning defects and a reduced expression of lysosomal markers [76].

Oxidation resistance 1 (OXR1) has emerged as a critical regulator of neuronal survival in response to oxidative stress [95,96,97]. A recent study showed that the OXR1 protein regulates an alternative pathway of v-ATPase holoenzyme disassembly [98]. This mechanism is ATP-independent and does not require the participation of the RAVE complex [75]. Loss-of-function in the gene’s *OXR1* variants was associated with cerebellar atrophy and other severe neurological conditions [99,100]. Consistent with these observations, neuron-specific depletion of *mtd*, the homolog of *NCOA7* and *OXR1* in flies, led to an aberrant lysosomal accumulation, massive neuronal loss, and early death [99]. *OXR1* patient-derived neurons displayed impaired neural differentiation and a dysregulated transcriptome [100]. Taken together, these studies show that aberrant mutations in TLDc proteins lead to impaired v-ATPase assembly and function, and ultimately to a wide spectrum of neurological conditions. However, due to the profound variability in clinical phenotypes, additional studies are needed to tease out the specific mechanisms at play.

## 4. Therapeutical Potential of V-ATPase Regulation in the Brain

### 4.1. Direct Modulation of the V-ATPase Activity or Expression

Modulating v-ATPase activity has previously shown therapeutic potential in a variety of disorders, ranging from cancer to viral infections [15]. V-ATPase also plays a role in protection against oxidative stress, a common feature in many neurodegenerative disorders [101]. Thus, developing targeted interventions on tissue-specific v-ATPases can represent a therapeutic option against disorders of different etiology.

To this end, Chung et al. discovered a small-molecule activator of autophagy, EN6, that acts through covalent targeting of a unique regulator to cysteine 277 of the ATP6V1A subunit, thus blocking mTORC1 lysosomal localization and activation [102]. In particular, the EN6-mediated ATP6V1A modification decouples v-ATPase from Rags, leading to inhibition of mTORC1 signaling. Notably, due to its high specificity for mTORC1, unlike other available drugs targeting the mTOR pathway, treatment with EN6 had no significant off-target effects on Akt signaling pathways [102]. Upon EN6 treatment TFEB transcriptional gene targets, including v-ATPase components were significantly increased thus promoting lysosomal acidification and autophagy progression. EN6 boosts cellular clearance of toxic protein aggregates in inducible Tar-binding protein 43 cells and in vivo treatment with EN6 significantly inhibited mTORC1 signaling in both skeletal muscle and the heart [102].

Direct enhancement of vATPase subunit gene expression represents an alternative and valid approach. For instance, Wang et al. demonstrated that the histone deacetylase inhibitor NCH-51 acts as a selective enhancer of *ATP6V1A* expression, thus normalizing neuronal dysfunction and neurodegeneration caused by loss of ATP6V1A [25]. In human-cultured *ATP6V1A*-silenced neurons, 24 h exposure to 3 μM NCH-51 was adequate to significantly increase ATP6V1A and its related subnetwork thus restoring neural excitability. Administration of 50 μM NCH-51 during aging in a Drosophila model genetically ablated for the two *ATP6V1A* orthologs, dampened Aß42-associated neurodegeneration, and partially increased the mRNA levels of synaptic genes, suggesting that NCH-51 confers neuroprotective effects by correcting neuronal activity [22].

In addition, a role for Rifampicin, an ansamycin antibiotic commonly used against tuberculosis, in the modulation of v-ATPase subunit expression has been proposed. In previous studies, Rifampicin was shown to have a neuro-protective function in acute and chronic brain injury by regulating inflammatory and the PI3K/Akt pathways [103,104,105]. More recently, Liang et al. showed that rifampicin inhibits rotenone-induced microglia inflammation via improving lysosomal function and autophagy clearance. This effect was mediated by a concomitant increase in the expression of *ATP6V0A1*, encoding the V0 subunit A [106]. Although the precise molecular mechanism linking rifampicin and ATP6V0A1 levels remains unknown, this finding may open an unexplored therapeutical potential for rifampicin in disorders associated with v-ATPase dysfunction.

Natural compounds have also been shown to modulate ATP6V1A activity. FK506 (also kwon as Tacrolimus) is a metabolite produced by the fungus *Streptomyces tsukbaenis*, which acts as strong immunosuppressor by inhibiting the calcium sensitive protein phosphatase calcineurin in T lymphocytes [107]. Kim et al. reported that FK506 selectively bound the V1A subunit and exerted neuroprotective effects by promoting autophagy following activation of the TFEB pathway [108]. Furthermore, dendrobium nobile Lindl. alkaloids (DNLAs), originally extracted from the traditional Chinese herbal medicine, *Dendrobium nobile*, displayed neuroprotective features and promoted autophagy in an animal model of axonal degeneration [109]. Nie et al. showed that DNLA improved lysosome acidification in the hippocampi of amyloid precursor protein/presenilin 1 (*APP/PS1*) mutant mice by increasing the expression of *ATP6V1A* [110].

In contrast with these studies, constitutive downregulation of *ATP6V1A* has been shown to be a potential therapeutical option in amyotrophic lateral sclerosis [111]. Treatment with the FDA-approved ATP6V1A blocker etidronate, a bisphosphonate largely used in osteoporosis treatment [112], decreased levels of the amyotrophic lateral sclerosis associated protein ataxin-2 in cellular models and brains of adult *Ataxin-2* mice [111]. Despite disagreeing with previous data linking lower ATP6V1A levels with pathogenicity [19,20,25,28,113], these results emphasize the importance of ATP6V1A as a therapeutic target for both novel and repositioned drugs, as its regulation is imperative to support neural function and survival. Early intervention on *ATP6V1A* expression and function could prevent neurodegeneration and promote synaptic integrity and plasticity in *ATP6V1A*-mediated disorders.

### 4.2. Indirect Strategies for Compensating V-ATPase Dysfunction

Contrasting the acidification defects associated with v-ATPase dysfunction may constitute an alternative therapeutical strategy for the here-described neurological disorders. For instance, EMD87580 a benzoyl-guanidine derivative, also known as rimeporide, is a potent inhibitor of the Sodium (Na^+^)/proton(H^+^) exchangers (NHE), which transport sodium in exchange for proton across lipid bilayers [114]. NHE channel isoforms could reside on the plasma membrane (NHE1–5) to maintain cytoplasmatic pH or at intracellular organelles (NHE6–9), thus regulating luminal acidification [115]. EMD87580, first described as selective blocker of NHE1 channel [116,117], has been reported as a strong antagonist of the endo-lysosomal NHE6 isoform [118]. Xian et al. showed that blocking NHE-6 with EMD87580 lowered endosomal pH by hindering proton leak in early endosomes. This, in turn, prevented the aggregation of the Alzheimer’s disease-associated Apolipoprotein E4 and restored physiological trafficking of ApoE receptor and AMPA- and NMDA-type glutamate receptors [118].

EMD87580 has a good safety and tolerability profile in adults and children and has been selected for different clinical phase I studies, including for congestive heart failure and Duchenne muscular dystrophy (DMD) [116]. Previtali et al. tested EMD87580 with the main goal of preventing or slowing the progression of myocardial damage in DMD children. Pharmacodynamic biomarkers analysis showed encouraging early changes of efficacy and biological effects after 4 weeks of treatment in 20 DMD male patients [116]. Furthermore, 24 h post-stroke EMD87580 treatment in rats, accelerated motor and cognitive function recovery and reduced microglial inflammatory activation and white matter demyelination [119], suggesting the efficacy of EMD87580 treatment also in neurological conditions.

The CIC-7 channel, belonging to the CIC family of chloride (Cl^−^) transport membrane proteins may represent an alternative route for contrasting v-ATPase-dependent deacidification. CIC7 is located to late endosomes and lysosomes where it allows the import of Cl^−^ ions and acts as counterion conductance channel to prevent hyperpolarization of the lysosomal membrane driven by v-ATPase-mediated proton import [120,121]. Lee and colleagues showed that clinically used agent isoproterenol (ISO), belonging to the β2-adrenergic receptor agonist family, normalized elevated pH in *PS1* KO cells and primary fibroblasts derived from *PS1* patients with FAD [122]. ISO is a synthetic catecholamine derived from noradrenaline and was commonly used as a bronchodilator before being dismissed because of its effect on cardiac β receptors [123]. In vitro, 6 h exposure to ISO stimulates lysosomal translocation of ClC-7 from endoplasmic reticulum thus normalizing Cl^−^ levels and upregulating lysosomal Cl^−^ channel function in these models. This, in turn, prevented lysosomal hydrolase impairment and autophagic failure restoring lysosomal proteolysis and calcium homeostasis in *PS1* in vitro models [122].

TMEM175 is a lysosomal membrane potassium (K^+^) channel, recently described as acting as proton channel at acidic pH and representing a proton leakage pathway thus regulating, in concert with v-ATPase, the maintenance of the correct acidic pH in lysosomal compartments [124]. Interestingly, numerous genetic studies have identified TMEM175 as a risk gene for Parkinson’s disease [125,126,127,128]. Exposure to the K^+^ channel blocker 4-aminopyridine and to high levels of Zn^2+^ have been shown to inhibit TMEM175 activity [129,130]; however, the associated undesirable effects mean that these approaches are not achievable. The design of drugs that selectively act on TMEM175 may represent a further opportunity to treat neurological disorders associated with v-ATPase dysfunction.

The use of nanomaterials as nanoparticles may offer an additional strategy to correct lysosomal pH in a v-ATPase-independent manner. Due to their nontoxic nature and their ability to be internalized by neurons [131], nanoparticles are considered effective and convenient routes for drug administration. FDA-approved poly (DL-lactide-co-glycolide) (PLGA) acidic nanoparticles (aNPs) have been successfully tested in different models of lysosomal disorders [132,133,134]. PLGA-aNP traffic to neuronal lysosomes within 24 h from intracerebral injection efficiently restored physiological pH in defective lysosomes, thus ameliorating PD-related dopaminergic neurodegeneration in vitro and in vivo [133]. PLGA-aNPs have also been tested in a *PS1* model, where they rescue lysosomal acidification and autophagy [135]. Lastly, in vivo administration of PLGA-aNPs can prevent α-syn-triggered dopaminergic neurodegeneration in PD mouse model [136]. PLGA-aNP can efficiently counteract lysosomal acidification impairment in fibroblasts of patients affected by X-linked myopathy with excessive autophagy, a genetic disorder related to VMA21, an essential chaperone of the v-ATPase, further strengthening the feasibility of an acidic nanoparticle-based strategy on this class of diseases [133,137].

Another intriguing therapeutical option could arise from recent studies connecting iron homeostasis with lysosomal dysfunction. Iron dyshomeostasis represents an early and crucial step in neurodegeneration, accompanied by accumulation of iron in different neuronal compartments [138,139]. Iron release from the lysosome to the cytoplasm requires acidic pH [140]. Within lysosomes, Fe^3+^ is reduced to Fe^2+^ by the ferric reductase, whose activity depends on acidic pH. Because of its reactivity, cytosolic Fe^2+^ is immediately chelated and excess cytoplasmic iron must be either removed via direct extrusion or neutralized by inclusion in ferritin molecules destined to lysosomal degradation [139]. Impaired lysosomal acidification mediated by both v-ATPase inhibition and genetic depletion of V-ATPase promotes bioavailable iron deficiency resulting in non-apoptotic cell death, mitochondrial dysfunction, and oxidative stress [140,141,142]. In addition, FAD-associated variants in the amyloid-β protein precursor lead to the inhibition of neuronal v-ATPase, with a subsequent decrease in the bioavailability of Fe^2+^ [143]. Yambire et al. showed that iron deficiency was also able to trigger inflammatory signaling and induce cellular stress response in a Pompe disease brain model and in vitro neuronal models [140]. Remarkably, these pro-inflammatory phenotypes were all corrected by increasing iron concentration in the diet [140], identifying the modulation of iron homeostasis as a potential therapeutic mechanism to contrast v-ATPase dysfunction. Furthermore, treatment with the antioxidant vitamin E (α-tocopherol) significantly reduced iron overload in neurological disorders and counteracted ferroptosis, a type of cell death caused by the accumulation of iron-mediated lipid superoxidation [144,145,146]. Interestingly, Luthy et al. showed that vitamin E treatment rescued behavioral defects in a knock-in model of *TBC1D24* [85], showing that vitamin E may represent a therapeutical co-adjuvant also in v-ATPase-related brain disorders.

**Figure 2:**
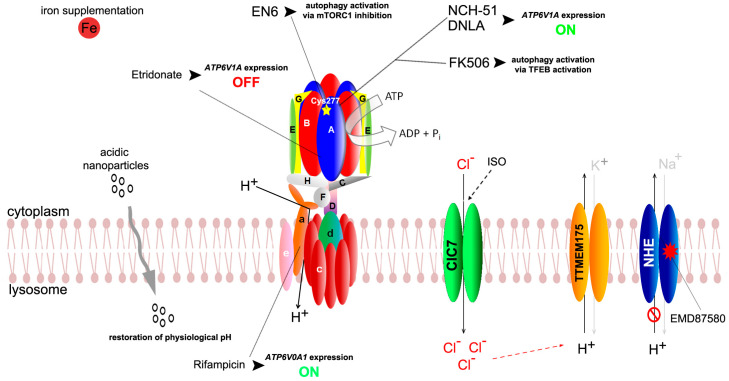
Schematic drawing depicting potential therapeutical approaches for v-ATPase-mediated brain disorders.

Taken together, these studies emphasize the potential role of selective approaches aiming to counteract lysosomal defects associated with v-ATPase dysfunction (Figure 2 and Table 2). 

## 5. Conclusions

**Figure 3:**
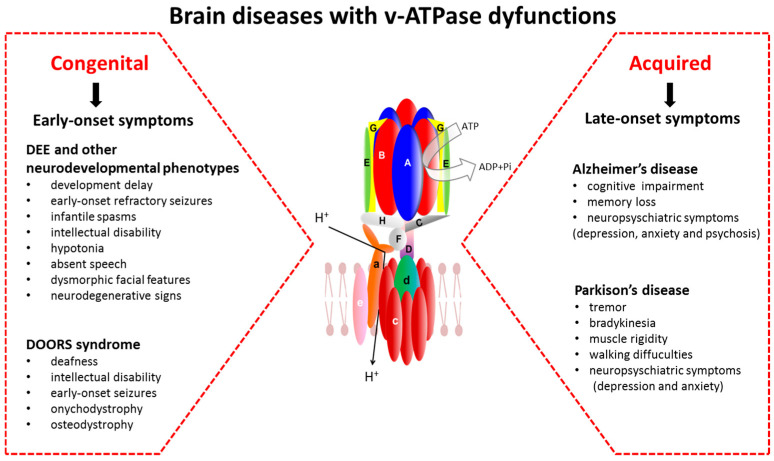
Overview of the clinical manifestations associated with congenital and acquired dysfunction of the v-ATPase in the brain. **Legend**: DEE, developmental and epileptic encephalopathy; DOORS, deafness, onychodystrophy, osteo-dystrophy, retardation and seizures.

Proton-translocating ATPases are involved in transmembrane transport, pH homeostasis, and lysosome acidification. v-ATPases are expressed at high levels in neurons, where they play additional roles, such as neurotransmitter loading into SVs [10,11]. Mutations in different v-ATPase domains and accessory proteins can disrupt proper assembly and function of the pump, leading to impaired cellular processes, such as small molecule transport, protein degradation, intracellular signaling, and autophagy. In the brain, these dysfunctions can also affect development and synaptic function, and contribute to encephalopathy and neurodegeneration in pre-clinical and human models [19,20,25,29,37,38,59,72,147,148,149] (Figure 3).

This work represents an up-to-date report of the characterized genetic variants of v-ATPase V0 and V1 domains, as well as their accessory proteins ATP6AP2, DMXL2, and TLDc domain-containing proteins. While v-ATPase dysfunction generally leads to pathological conditions, the specific cognitive and neuropathological phenotypes vary among different variants. Further studies are necessary to elucidate the precise mechanisms involved. A deeper understanding of the role of this proton pump in the brain and of its pathogenic variants will aid in the development of precision medicine approaches to improve the clinical symptoms and quality of life in patients with neurological disorders related to v-ATPase dysfunction. Promising potential strategies are now emerging, and future studies will make it possible to define the most appropriate, safe, and effective delivery methods.

## Figures and Tables

**Table 1:** Synopsis of brain disorders related to genetic v-ATPase dysfunction. Abbreviations: AD, autosomal dominant; AR, autosomal recessive; XLR, X-linked recessive; DEE, developmental and epileptic encephalopathy; DOORS, deafness, onychodystrophy, osteodystrophy, retardation and seizures; ZLS2, Zimmermann–Laband syndrome 2; NDD, neurodevelopmental disorder; EEO3, early-onset epilepsy 3; XLID, X-linked intellectual disability; XPDS, X-linked parkinsonism with spasticity; FIME, familial infantile myoclonic epilepsy; EPRPDC.; epilepsy rolandic with paroxysmal exercise-induced dystonia and writer’s cramp; CHEGDD, cerebellar hypoplasia/atrophy, epilepsy, and global developmental delay; ASD, autism spectrum disorder.

Human Gene (NCBI Ref.Seq)	Gene Product	Inheritance	Associated Neurogenetic Conditions (OMIM)	Main and Clinical Manifestations
*ATP6V1A* (NM_001690.4)	V1 subunit A	AD	DEE 93 (OMIM #618012)	delayed psychomotor development; early-onset refractory seizures; impaired intellectual development; cerebral and cerebellar atrophy; progressive neurodegeneration
*ATP6V1B2* (NM_001693.3)	V1 subunit B	AD	DOORS syndrome (OMIM #220050); ZLS2 syndrome (OMIM #616455); DEE.; Autosomal dominant epilepsy with or without intellectual disability	delayed psychomotor development; early-onset refractory seizures; impaired intellectual development; cerebral and cerebellar atrophy; progressive neurodegeneration; specific dysmorphic features (DOORS patients)
*ATP6V0A1* (NM_001130020.3)	V0 subunit A	AD/AR	DEE 104 (OMIM #619970); NDDs with epilepsy and brain atrophy (OMIM #619971)	delayed psychomotor development; early-onset refractory seizures; impaired intellectual development; cerebral and cerebellar atrophy; progressive myoclonus epilepsy with ataxia
*ATP6V0C* (NM_001694.4)	V0 subunit C	AD	EEO3, with or without developmental delay (OMIM #620465)	delayed psychomotor development; early-onset refractory seizures; impaired intellectual development; gait ataxia, nonspecific dysmorphic features
*ATP6AP2* (NM_005765.3)	ATPase, H+ transporting lysosomal accessory protein 2	XLR	XLID, epilepsy and neurodegeneration (OMIM #300423); XPDS (OMIM #300911)	parkinsonian features and spasticity; delayed psychomotor development; early-onset refractory seizures; impaired intellectual development; cerebral and cerebellar atrophy; progressive neurodegeneration
*DMXL2* (NM_001174116.3)	Rabconnectin-3α	AR	DEE 81 (OMIM #618663)	delayed psychomotor development; early-onset refractory seizures; impaired intellectual development; cerebral and cerebellar atrophy; progressive neurodegeneration; facial dysmorphism; progressive leukoencephalopathy
*TBC1D24* (NM_001199107.2)	TBC1 Domain Family Member 24	AR	FIME (OMIM #605021); DEE 16 (OMIM #615338); DOORS syndrome (OMIM# 220050); EPRPDC (OMIM# 608105)	delayed psychomotor development; early-onset refractory seizures; impaired intellectual development; myoclonic epilepsy (FIME patients), specific dysmorphic features (DOORS patients); exercise-induced dystonia (EPRPDC patients)
*OXR1* (NM_001198532.1)	Oxidation Resistance 1	AR	CHEGDD (OMIM# 213000)	delayed psychomotor development; impaired intellectual development; seizures; cerebellar atrophy and dysplasia; ataxia; nonspecific dysmorphic features
*NCOA7* (NM_001199619.2)	Nuclear receptor coactivator 7	AR	ASD	autistic features

**Table 2:** Potential therapeutical approaches discussed in the study.

Treatment	Target	Mechanism(s) of Action	Tested Models	Ref
EN6	ATP6V1A	Covalent targeting of cysteine 277 of the ATP6V1A subunit and enhancement of autophagy as consequence of mTORC1 inactivation	Mouse and human cells lines; skeletal muscle and heart mouse tissues	[102]
NCH-51	ATP6V1A	Histone deacetylase inhibitor, selective enhancement of *ATP6V1A* expression	Drosophila model and human-induced pluripotent stem cell-derived neurons	[25]
Rifampicin	ATP6V0A1	Enhancement of ATP6V0A1 expression	Immortalized human microglia cells	[106]
FK506 (Tacrolimus)	ATP6V1A	Binding with ATP6V1A followed by TFEB nuclear translocation and activation of autophagy	SH-SYSY neuroblastoma cell line	[108]
*Dendrobium nobile Lindl. alkaloids*	ATP6V1A	Increased ATP6V1A expression, improved lysosome acidification, and autophagy	amyloid-β and APP/PS1 mice mouse models	[109,110]
Etidronate	ATP6V1A	ATP6V1A inhibition	*Ataxin-2* mouse model	[111]
EMD87580 (rimeporide)	NHE6 channel	Contrasting lysosomal deacidification by preventing proton efflux at the endolysosome	Animal models of myocardial infarction, dystrophic cardiomyopathy, and DMD models; phase I clinical trials in DMD patients	[116,117,118,119]
Isoproterenol (ISO)	CIC-7 channel	Contrasting lysosomal deacidification by enhancing parallel Cl^−^ conductance at the lysosome	*PS1* KO cells; primary fibroblasts derived from *PS1* patients with familial AD	[122]
Poly(DL-lactide-co-glycolide) acidic nanoparticles (PLGA-aNP)	Lysosomes	Direct lowering of lysosomal pH after internalization	Human fibroblasts; BE(2)-M17 neuroblastoma cell line; mouse brain tissues, injected with PD patient-derived Lewy body extracts	[133,135,136,137]
Iron supplementation	Cellular iron homeostasis	Contrasting bioavailable iron deficiency associated with v-ATPase dysfunction	Yeast lacking v-ATPase components; HEK293T depleted for *ATP6V0C.; Gaa-*KO mice	[140,141,142]
